# Relationship between pulmonary function and albuminuria in type 2 diabetic patients with preserved renal function

**DOI:** 10.1186/s12902-020-00598-1

**Published:** 2020-07-23

**Authors:** Yun-Yun He, Zhe Chen, Xiang-Yang Fang, Jing Chang, Yong Lu, Xiao-Juan Wang

**Affiliations:** 1 Department of General Internal Medicine, Beijing Chao-Yang Hospital, Capital Medical University, Beijing, 100020 China; 2 Department of Endocrinology, Beijing Chao-Yang Hospital, Capital Medical University, Beijing, 100020 China; 3 Department of Respiratory and Critical Care Medicine, Beijing Chao-Yang Hospital, Capital Medical University, Beijing, 100020 China

**Keywords:** Type 2 diabetes mellitus, Pulmonary function, Albuminuria, Preserved renal function

## Abstract

**Background:**

Albuminuria is the early manifestation of the pathogenesis of diabetic nephropathy (DN). The current study was to investigate the relationship of pulmonary function with albuminuria in type 2 diabetic patients with preserved renal function to evaluate the role of pulmonary function in the early stage of DN.

**Methods:**

A total of 326 patients with type 2 diabetes mellitus (T2DM) including 270 without albuminuria and 56 with albuminuria, and 265 non-diabetic patients were enrolled. The patients’ general information, and the parameters of pulmonary function, including forced vital capacity (FVC), forced expiratory volume in 1 s (FEV1), FEV1/FVC, total lung capacity (TLC), diffusion capacity for carbon monoxide of lung (DLCO) were compared between T2DM and control groups, as well as T2DM patients with and without albuminuria groups. All pulmonary function parameters were expressed as a percentage of those predicted (%pred). Logistic regression models were constructed to test the association of albuminuria and pulmonary function.

**Results:**

The values of FVC%pred, FEV1%pred, TLC%pred and DLCO%pred were lower, and the proportion of subjects with FVC%pred < 80, FEV1%pred < 80, and DLCOc%pred < 80 was higher in T2DM subjects than controls (all *P* < 0.05). Subgroup analysis of diabetic patients showed that the values of FVC%pred, FEV1%pred, TLC%pred, and DLCOc%pred (97.18 ± 13.45, 93.95 ± 14.51, 90.64 ± 9.97, 87.27 ± 13.13, respectively) were significantly lower in T2DM subjects with albuminuria than those without albuminuria (103.94 ± 14.12, 99.20 ± 14.25, 93.79 ± 10.36, 92.62 ± 13.45, all *P* < 0.05). There was a significantly negative correlation between the urine albumin-to-creatinine ratio (UACR) and DLCOc%pred (*r* = − 0.143, *P* = 0.010) in spearman linear correlation test. In logistic regression analysis, the FVC%pred (OR 0.965, 95%CI 0.944–0.988), FEV1%pred (OR 0.975, 95%CI 0.954–0.996), and DLCOc%pred (OR 0.974, 95%CI 0.951–0.998) were independently associated with albuminuria after adjustments for smoking index, duration, HbA1c, FBG, and TG.

**Conclusion:**

Our results demonstrated albuminuria is associated with a restrictive pulmonary function as well as pulmonary diffusion function in T2DM with preserved renal function, which remind us to be alert of the pulmonary function decline even in the early stage of DN.

## Background

Type 2 diabetes mellitus (T2DM) is a chronic disease characterized by glucose metabolism disorder. As the disease progresses, it can cause microvascular and macrovascular complications involving kidney, retina, nerve and cardiovascular system. Although the lung has not been listed as a classical target organ for diabetes, the abundant pulmonary alveolar capillary network and connective tissues raise the possibility that the lung may also be affected by chronic hyperglycemia [1]. To date, the findings in the clinical studies about the relationship between pulmonary function and T2DM are controversial. Some studies believe that the clinical value is not significant, and routine pulmonary function testing is not recommended in patients with diabetes [2, 3]. However, more studies support that T2DM has a deleterious effect on pulmonary function [4–6].

Diabetic nephropathy (DN) is one of the most important microvascular complications in T2DM. It’s usually diagnosed in the middle to late stages when the renal function is declined. Albuminuria, as indicated by the urine albumin-to-creatinine ratio (UACR), is the first manifestation of the pathogenesis of DN even with preserved renal function [7]. Additionally, it is also an independent risk factor of cardiovascular events and mortality [8]. Although previous studies have identified a relationship between pulmonary function and UACR as well as chronic kidney disease with reduced estimated glomerular filtration rate (eGFR) [9–11], few studies focus on the T2DM patients with preserved eGFR. As we know, those with T2DM are more likely to have albuminuria and at a higher risk for cardiovascular disease even with preserved eGFR [12]. However, the relationship between the pulmonary function and albuminuria in these groups is unclear.

The aim of our study was to investigate the relationship between pulmonary function and albuminuria in T2DM patients with preserved renal function. Such information would be useful to evaluate the changes of pulmonary function in the early stage of DN.

## Methods

### Subjects

Three hundred and twenty-six patients with T2DM who were admitted to Department of General Internal Medicine and Endocrinology of Beijing Chao-Yang Hospital from January 2018 to December 2019 were included in this study. The diagnosis of T2DM was according to the criteria of World Health Organization 1999 or treatment with insulin or other hypoglycemic agents. Also, 265 subjects without T2DM were included as controls.

The exclusion criteria were acute infections, renal dysfunction (eGFR < 60 ml/min/1.73m^2^), other kidney diseases, occupational dust exposure history, acute and chronic pulmonary basic diseases, cardiac insufficiency, malignant tumors, acute coronary syndrome and acute cerebrovascular diseases within 3 months, thoracic malformation, connective tissue diseases, blood system diseases and acute diabetic ketoacidosis. All subjects underwent biochemistry, chest x-ray or CT, electrocardiogram, and echocardiography to exclude the above conditions. The study was approved by the Ethical Committee for Medical Research of Beijing Chao-Yang Hospital and accorded with Helsinki Declaration. Informed consent was obtained from all subjects.

### Clinical data collection

The clinical parameters of the patients, including sex, age, diabetes duration, and smoking status, were collected in detail by face-to-face interview. The height and weight of patients was measured in pulmonary function laboratory by the same gauges. The Body mass index (BMI) was calculated by dividing the weight (in kilograms) by the height (in meters) squared. Smoking status included current smoking, smoking cessation for more than 1 year, and never smoking. Smoking index was defined as the average root number per day multiplied by smoking years.

Biochemical variables were included glycated hemoglobin A1c (HbA1c), fasting blood glucose (FBG), low-density lipoprotein cholesterol (LDL), high-density lipoprotein cholesterol (HDL), triglyceride level (TG) and serum creatinine. The eGFR was calculated by Chronic Kidney Disease Epidemiology Collaboration (CKD-EPI) formula [13].

The random urine albumin and urine creatinine were determined by nephelometry in the clinical laboratory. The incident albuminuria is defined as the UACR ≥30 mg/g.

### Pulmonary function testing

Spirometry was measured by experienced physicians using Jaeger MasterScreen pulmonary function instrument and all tests were performed according guidelines of pulmonary function professional group of respiratory branch of Chinese medical association. The examination parameters included forced vital capacity (FVC), forced expiratory volume in first second (FEV1), FEV1/FVC, total lung capacity (TLC), diffusion capacity for carbon monoxide (DLCO), alveolar volume (VA) and DLCO/VA. All subjects were required to provide at least three acceptable spirometry curve and 150 ml intermeasurement variability in FVC and FEV1 [14]. DLCO was measured at least twice with a 5-min snooze and 10% intermeasurement variability [15]. All values were expressed as a percentage of those predicted (%pred) for age, sex, height and weight based on data from Chinese general population.

### Statistical analysis

All tests were carried out using SPSS version 22.0 statistical software. The continuous variables were shown as mean ± standard deviation or median (minimum, maximum). Statistical analysis of continuous variables was performed using an unpaired Student t test or Mann-Whitney U test between two groups, and by one-way analysis of variance (ANOVA) or Kruskal-Wallis for multiple comparisons. Categorical variables were expressed as frequency and percentages, and performed by chi-square test or Fisher exact test. Spearman linear correlation test was employed to study the association between UACR and pulmonary function parameters. Multivariate logistic regression analysis with forward stepwise method was performed to evaluate the association between pulmonary function and albuminuria, by adjustment the parameters with a *P* value of less than 0.05 in the univariate analysis. Differences were considered statistically significant at a two-tailed *P* value < 0.05.

## Results

### Clinical characteristics and pulmonary function in T2DM and control subjects

A total of 326 T2DM participants and 265 controls were included in this study. The characteristics of T2DM and controls are listed in Table 1. There were no statistically significant differences in sex and age. Patients with T2DM had significant higher BMI (27.07 ± 3.86 vs 26.31 ± 3.63 kg/m^2^, *P* = 0.015), lower median smoking index (40 vs 300, *P* = 0.003), less current smokers (42% vs 54%, *P* = 0.001) and higher eGFR (105.46 ± 14.20 vs 100.89 ± 11.48 ml/min/1.73m^2^, *P* = 0.000) than controls.

**Table 1:** Demographic and clinical characteristics of control and T2DM subjects

	Controls	T2DM	*P*
	(*n* = 265)	(*n* = 326)	
Sex			
Male, n (%)	192(72.5)	222(68.1)	0.250
Female, n (%)	73(27.5)	104(31.9)	
Age	52.55 ± 10.11	53.52 ± 11.57	0.282
BMI (kg/m^2^)	26.31 ± 3.63	27.07 ± 3.86	0.015
Smoking index	300(0, 2400)	40(0, 2400)	0.003
Current smoking status			
Never, n (%)	87(32.8)	156(47.9)	0.001
Former, n (%)	35(13.2)	33(10.1)	
Current, n (%)	143(54.0)	137(42.0)	
HbA1c (%)	5.54 ± 0.29	9.07 ± 2.22	0.000
FBG (mmol/L)	4.91 ± 0.67	7.65 ± 2.66	0.000
HDL (mmol/L)	1.15 ± 0.32	1.02 ± 0.25	0.000
LDL (mmol/L)	2.82 ± 0.80	2.78 ± 0.92	0.580
TG (mmol/L)	2.01 ± 1.44	2.16 ± 1.58	0.242
eGFR (ml/min/1.73m^2^)	100.89 ± 11.48	105.46 ± 14.20	0.000

Data are presented as mean ± standard deviation, median (minimum, maximum), or number with percentage (%)

*BMI* body mass index, *HbA1c* glycated hemoglobin A1c, *FBG* fasting blood glucose, *LDL* low-density lipoprotein cholesterol, *HDL* high-density lipoprotein cholesterol, *TG* triglyceride level, *eGFR* estimated glomerular filtration rate

The pulmonary function tests showed that in T2DM patients, the values of FVC%pred, FEV1%pred, TLC%pred, and DLCOc%pred were (102.78 ± 14.22, 98.30 ± 14.41, 93.24 ± 10.34, 91.7 ± 13.53, respectively) significantly lower than those in controls (107.21 ± 13.05, 101.56 ± 12.67, 97.48 ± 10.17, 94.80 ± 12.83, all *P* < 0.05). In contrast, the FEV1/FVC% was higher in T2DM patients than controls (78.37 ± 5.46 vs 77.49 ± 4.88, *P* = 0.043). We also found that the proportion of subjects with FVC%pred < 80, FEV1%pred < 80, and DLCOc%pred < 80 was higher in T2DM than controls (4.0% vs 0.8%, 9.8% vs 2.6%, 16.6% vs 9.8%, all *P* < 0.05). There was no statistically difference in DLCOc/VA%pred. (Table 2).

**Table 2:** Pulmonary function data of control and T2DM subjects

	Control	T2DM	*P*
	(*n* = 265)	(*n* = 326)	
FVC%pred	107.21 ± 13.05	102.78 ± 14.22	0.000
< 80, n (%)	2 (0.8)	13 (4.0)	0.026
> 80, n (%)	263 (99.2)	313 (96.0)	
FEV1%pred	101.56 ± 12.67	98.30 ± 14.41	0.004
< 80, n (%)	7(2.6)	32 (9.8)	0.001
> 80, n (%)	258 (97.4)	294 (90.2)	
FEV1/FVC (%)	77.49 ± 4.88	78.37 ± 5.46	0.043
TLC%pred	97.48 ± 10.17	93.24 ± 10.34	0.000
DLCOc%pred	94.80 ± 12.83	91.70 ± 13.53	0.005
< 80, n (%)	26 (9.8)	54 (16.6)	0.023
> 80, n (%)	239 (90.2)	272 (83.4)	
DLCOc/VA%pred	99.72 ± 14.06	101.61 ± 15.04	0.119

Data are presented as mean ± standard deviation, or number with percentage (%)

*FVC* forced vital capacity, *FEV1* forced expiratory volume in 1 second, *TLC* total lung capacity, *DLCO* diffusion capacity for carbon monoxide of lung, *VA* alveolar volume

The results of a sub-analysis by smoking status showed that the values of FVC%pred, FEV1%pred, and TLC%pred were significantly lower in T2DM than those in controls for both never smokers (105.48 ± 15.14 vs 112.72 ± 14.09, 100.86 ± 14.74 vs 106.55 ± 14.36, 93.89 ± 11.00 vs 99.40 ± 11.04, all *P* < 0.05) and former/current smokers (100.29 ± 12.86 vs 104.52 ± 11.63, 95.95 ± 13.73 vs 99.13 ± 11.00, 92.66 ± 9.71 vs 96.55 ± 9.60, all *P* < 0.05). Also, in the subgroup of never smokers, the DLCOc%pred of T2DM was (91.39 ± 13.53 vs 97.03 ± 14.91, *P* < 0.05) significantly lower than those in controls. In addition, the FEV1/FVC% was highest in T2DM of never smokers and lowest in controls of former/current smokers (79.21 ± 5.18 vs 76.99 ± 4.83, *P* < 0.05). (Table S1).

### The subgroup analysis of clinical characteristics and pulmonary function in T2DM subjects with or without albuminuria

There were 270 T2DM patients without albuminuria (UACR < 30 mg/g) and 56 with albuminuria (UACR ≥30 mg/g) in this study. No statistically significant differences were found in sex, age, BMI, and smoking status between the two groups. The T2DM patients with albuminuria had longer median duration (7.5 vs 5, *P =* 0.032), higher HbA1c (9.69 ± 2.58 vs 8.94 ± 2.12, *P* = 0.021), FBG (8.60 ± 3.22 vs 7.46 ± 2.49 mmol/L, *P* = 0.003) and TG (2.86 ± 2.14 vs 2.02 ± 1.39 mmol/L, *P* = 0.000) than those without albuminuria. (Table 3).

**Table 3:** Clinical characteristics of T2DM subjects without or with albuminuria

	Without albuminuria	Albuminuria	*P*
	(*n* = 270)	(*n* = 56)	
Sex			
Male, n (%)	182(67.4)	40(71.4)	0.557
Female, n (%)	88(32.6)	16(28.6)	
Age	53.72 ± 11.46	52.54 ± 12.13	0.487
BMI (kg/m^2^)	26.97 ± 3.97	27.55 ± 3.89	0.308
Smoking index	18.5(0, 2400)	112.5(0, 2400)	0.141
Current smoking status			
Never, n (%)	131(48.5)	25(44.7)	0.508
Former, n (%)	29(10.7)	4(7.1)	
Current, n (%)	110(40.8)	27(48.2)	
Duration (year)	5(0, 30)	7.5(0, 41)	0.032
HbA1c (%)	8.94 ± 2.12	9.69 ± 2.58	0.021
FBG (mmol/L)	7.46 ± 2.49	8.60 ± 3.22	0.003
HDL (mmol/L)	1.02 ± 0.24	1.01 ± 0.29	0.857
LDL (mmol/L)	2.76 ± 0.90	2.87 ± 1.03	0.450
TG (mmol/L)	2.02 ± 1.39	2.86 ± 2.14	0.000
eGFR (ml/min/1.73m^2^)	105.48 ± 13.89	105.37 ± 15.76	0.955

Data are presented as mean ± standard deviation, median (minimum, maximum), or number with percentage (%)

*BMI* body mass index, *HbA1c* glycated hemoglobin A1c, *FBG* fasting blood glucose, *LDL* low-density lipoprotein cholesterol, *HDL* high-density lipoprotein cholesterol, *TG* triglyceride level, *eGFR* estimated glomerular filtration rate

The values of FVC%pred, FEV1%pred, TLC%pred, and DLCOc%pred were (97.18 ± 13.45, 93.95 ± 14.51, 90.64 ± 9.97, 87.27 ± 13.13, respectively) significantly lower in T2DM with albuminuria than those without albuminuria (103.94 ± 14.12, 99.20 ± 14.25, 93.79 ± 10.36, 92.62 ± 13.45, all *P* < 0.05). There were no significant differences in FEV1/FVC% and DLCOc/VA%pred between the two groups. The proportion of subjects with FVC%pred < 80, FEV1%pred < 80, and DLCOc%pred < 80 had an increasing tendency in albuminuria group, but no statistical significance (5.4% vs 3.7%, 16.1% vs 8.5%, 25% vs 14.8%, all *P* > 0.05). (Table 4).

**Table 4:** Pulmonary function data of T2DM subjects without or with albuminuria

	Without albuminuria	Albuminuria	*P*
	(*n* = 270)	(*n* = 56)	
FVC%pred	103.94 ± 14.12	97.18 ± 13.45	0.001
< 80, n (%)	10 (3.7)	3 (5.4)	0.841
> 80, n (%)	260 (96.3)	53 (94.6)	
FEV1%pred	99.20 ± 14.25	93.95 ± 14.51	0.013
< 80, n (%)	23 (8.5)	9 (16.1)	0.084
> 80, n (%)	247 (91.5)	47 (83.9)	
FEV1/FVC (%)	78.28 ± 5.28	78.78 ± 6.31	0.584
TLC%pred	93.79 ± 10.36	90.64 ± 9.97	0.038
DLCOc%pred	92.62 ± 13.45	87.27 ± 13.13	0.007
< 80, n (%)	40 (14.8)	14 (25.0)	0.062
> 80, n (%)	230 (85.2)	42 (75.0)	
DLCOc/VA%pred	101.61 ± 14.91	101.59 ± 15.80	0.992

Data are presented as mean ± standard deviation, or number with percentage (%)

*FVC* forced vital capacity, *FEV1* forced expiratory volume in 1 second, *TLC* total lung capacity, *DLCO* diffusion capacity for carbon monoxide of lung, *VA* alveolar volume

Moreover, in the sub-analysis by smoking status, T2DM with albuminuria had higher FBG than those without albuminuria for never smokers. The FVC%pred, FEV1%pred, and TLC%pred were highest in group of never smokers-without albuminuria and lowest in former/current smokers-albuminuria. The FVC%pred was significantly lower in T2DM with albuminuria than those without albuminuria for both never smokers (99.84 ± 13.80 vs 106.56 ± 15.20, *P* < 0.05) and former/current smokers (95.05 ± 12.99 vs 101.46 ± 12.58, *P* < 0.05). For former/current smokers, the DLCOc%pred of T2DM with albuminuria was lower than those without albuminuria (87.23 ± 12.13 vs 93.05 ± 13.68, *P* < 0.05). (Table S2).

### Correlation of UACR levels with pulmonary function

The correlation of UACR levels with pulmonary function parameters in T2DM subjects was analyzed by spearman linear correlation test. The results are shown in Table 5. There was a significantly negative correlation between UACR and DLCOc%pred (*r* = − 0.143, *P* = 0.010). There were no statistical correlation between UACR and FVC%pred as well as TLC%pred at a *P* value of 0.069 and 0.058, respectively.

**Table 5:** Correlation between pulmonary function and UACR in T2DM Group

	*r*	*P*
FVC%pred	−0.101	0.069
FEV1%pred	−0.090	0.105
FEV1/FVC(%)	0.020	0.714
TLC%pred	−0.105	0.058
DLCOc%pred	−0.143	0.010
DLCOc/VA%pred	−0.025	0.651

*FVC* forced vital capacity, *FEV1* forced expiratory volume in 1 second, *TLC* total lung capacity, *DLCO* diffusion capacity for carbon monoxide of lung, *VA* alveolar volume

### Multivariate logistic regression analyses for albuminuria

The logistic regression analysis was performed to study the independent associations with albuminuria in T2DM patients (Table 6). We analyzed sex, age, BMI, smoking index, duration, HbA1c, FBG, HDL, LDL, TG, eGFR, FVC%pred, FEV1%pred, FEV1/FVC, TLC%pred, DLCOc%pred, and DLCOc/VA%pred in the univariate logistic regression analysis. The *P* values of variables including smoking index, duration, HbA1c, FBG, TG, FVC%pred, FEV1%pred, TLC%pred, and DLCOc%pred were less than 0.05.

**Table 6:** The univariate and multivariate logistic regression analyses for albuminuria

	Univariate		Model1		Model2		Model2	
	OR (95% CI)	*P*	OR (95% CI)	*P*	OR (95% CI)	*P*	OR (95% CI)	*P*
Smoking index	1.001(1.000–1.001)	0.040						
Duration	1.053(1.015–1.096)	0.006	1.064(1.021–1.109)	0.003	1.065(1.022–1.109)	0.003	1.061(1.019–1.105)	0.004
HbA1c	1.156(1.021–1.309)	0.022	1.238(1.074–1.429)	0.003	1.228(1.068–1.413)	0.004	1.224(1.065–1.406)	0.004
FBG	1.155(1.046–1.276)	0.004						
TG	1.314(1.120–1.541)	0.001	1.365(1.147–1.624)	0.000	1.357(1.143–1.610)	0.000	1.330(1.1267–1.571)	0.001
FVC%pred	0.965(0.944–0.986)	0.001	0.965(0.944–0.988)	0.002				
FEV1%pred	0.974(0.954–0.995)	0.014			0.975(0.954–0.996)	0.022		
DLCOc%pred	0.969(0.948–0.992)	0.008					0.974(0.951–0.998)	0.035

*HbA1c* glycated hemoglobin A1c, *FBG* fasting blood glucose, *TG* triglyceride level, *FVC* forced vital capacity, *FEV1* forced expiratory volume in 1 second, *DLCO* diffusion capacity for carbon monoxide of lung, *OR* odds ratio, *CI* confidence interval

Model 1: included FVC%pred, smoking index, duration, HbA1c, FBG, and TG

Model 2: included FEV1%pred, smoking index, duration, HbA1c, FBG, and TG

Model 3: included DLCOc%pred, smoking index, duration, HbA1c, FBG, and TG

In the subsequent multivariate logistic regression models, we respectively explored whether the FVC%pred (model 1), FEV1%pred (model 2), DLCOc%pred (model 3), were independent associations with albuminuria, after adjustments for smoking index, duration, HbA1c, FBG, and TG. The results revealed that FVC%pred (OR = 0.965, 95% CI = 0.944–0.988, *P* = 0.002), FEV1%pred (OR = 0.975, 95% CI = 0.954–0.996, *P* = 0.022), and DLCOc%pred (OR = 0.974, 95% CI = 0.951–0.998, *P* = 0.035) were independently associated with albuminuria.

## Discussion

In the present study, our results verified the point that T2DM exerts a negative effect on pulmonary function manifested as a change of restrictive pulmonary ventilation and diffusion function. We also demonstrated the relationship between the pulmonary function and albuminuria in T2DM with preserved eGFR. We found that FVC%pred, FEV1%pred, and DLCOc%pred were independently associated with albuminuria after adjustments for smoking index, diabetic duration, HbA1c, FBG, and TG.

As a systemic disease, the opinions about the effect of diabetes on pulmonary function are divided. Analysis of current studies shows that most scholars support the existence of pulmonary function changes in diabetic patients, and put forward the concept of “diabetic lung” [16, 17]. The ARIC study including 1100 diabetics and 10,162 controls pointed out that abnormalities in pulmonary function, particularly vital capacity, precede diabetes, then continue after diabetes onset [4]. Also, studies reported that the impairment of diabetic pulmonary function is more reflected in the decline of diffusion function [18, 19]. In addition, a systematic review of 34 articles showed that adults with diabetes mellitus have lower FVC and FEV1, with reductions in FVC more consistent than FEV1 and lower DLCO compared with their non-diabetic counterpart [20]. Recently, a study by Kopf et al. found that diabetic patients are prone to interstitial lung disease [21]. Even in the prediabetes stage, the deleterious effect of T2DM on pulmonary function appears to be initiated [22].

It is generally known that smoking is one of the most critical influences on pulmonary function. In our study, compared with controls, the smoking index and the percentage of current smoking status were both lower in T2DM. Even so, the values of FVC%pred, FEV1%pred, TLC%pred, and DLCOc%pred were significantly lower in T2DM than controls. We further found that the proportion of subjects with FVC%pred < 80, FEV1%pred < 80, and DLCOc%pred < 80 was higher in T2DM than controls. In contrast, because of the smaller magnitude for FEV1 than FVC, there was an inversion of FEV1/FVC% in T2DM patients. These results indicated the changes of restrictive pulmonary ventilation and diffusion function in T2DM patients, which are generally consistent with majority of prior studies. In order to further get rid of the effect of smoking on pulmonary function, we then provided a sub-analysis by smoking status. The results also revealed the negative effect of T2DM on pulmonary function in both never smokers and former/current smokers. Also, the FEV1/FVC% was highest in T2DM of never smokers and lowest in controls of former/current smokers, which indicated the more important role of smoking in obstructive pulmonary function. Recently, a prospective study including 7524 participants also reported that diabetes is not associated with obstructive pulmonary function impairment [6]. In addition, some studies with small sample size reported that not only DLCOc%pred but also DLCOc/VA%pred was decreased in T2DM [23, 24]. However, in current study, there was no statistically difference in DLCOc/VA%pred, which may be associated with a decrease in alveolar ventilation (VA) induced by restrictive ventilation dysfunction.

Albuminuria is the best available surrogate parameter in the pathogenesis of DN, which is rised before the decrease of eGFR. On the contrary, the glomerular hyperfiltration is accured in the early stage of DN [25]. As described in this paper, the eGFR was higher in T2DM subjects than controls. Lee reported that abnormal pulmonary function is associated with increased odds for chronic kidney disease indicators, such as lower eGFR or albuminuria in metabolic syndrome [26]. It is also reported that pulmonary dysfunction, particularly restrictive dysfunction associates with degree of renal function impairment and contributes to the progression of chronic kidney disease [27, 28]. But, it is still unclerar whether the pulmonary function is declined or not in DN patients with preserved eGFR. Then, we explored the relationship between pulmonary function with albuminuria in T2DM patients with preserved eGFR, in order to make clear the changes of pulmonary function in the early stage of DN.

In the current study, there was no significant difference of eGFR between T2DM patients with or without albuminuria. The FVC%pred, FEV1%pred, TLC%pred, and DLCOc%pred were significantly lower in T2DM subjects with albuminuria than those without albuminuria. The sub-analysis by smoking status revealed that whether you smoke or not, the presence of albuminuria adversely affected the pulmonary function, especially more so with smokers. The spearman linear correlation test showed that there was a significantly negative correlation between UACR and DLCOc%pred. By multivariate logistic regression, we found that FVC%pred, FEV1%pred, and DLCOc%pred were independently associated with albuminuria after adjustments for smoking index, duration, HbA1c, FBG, and TG.

Albuminuria, which indicates that blood albumin has leaked from blood vessels of the kidneys into the urine, is a strong marker of microvascular damage. Hence, our current finding of an inverse relationship between pulmonary function and UACR is consistent with the evidence that pulmonary microangiopathy is detectable in T2DM subjects [29, 30]. These results remind us even in the early stage of DN, we should be alert of the decline of pulmonary function.

However, the proportion of subjects with FVC%pred < 80, FEV1%pred < 80, and DLCOc%pred < 80 in albuminuria subjects were increased but insufficient to reach statistical significance in the current study. As we know, the pulmonary alveolus-capillary network is the largest microvascular bed with powerfully physiological reserve in the human body [31]. So the change of pulmonary function of T2DM subjects in the early stage of DN is more marked by decline rather than dysfunction. Even so, we still think that it is necessary to carried out pulmonary function tests in T2DM patients whether you smoke or not, especially for those with albuminuria or other microangiopathy [32].

There are some limitations to our study. Firstly, this study was a single-center, cross-sectional study. Therefore, it was not possible to assess changes of pulmonary function over time and the implications of these changes on the T2DM outcomes. Secondly, the number of cases with albuminuria was relatively small, which did not allow subgroup analyses by microalbuminuria and proteinuria. A large-scale investigation about pulmonary function and albuminuria needs to be built to further confirm the relationship.

## Conclusions

In conclusions, our results verified the point that T2DM exerts a negative effect on pulmonary function manifested as a change of restrictive pulmonary ventilation and diffusion function. We also demonstrated that albuminuria is associated with a restrictive pulmonary function as well as pulmonary diffusion function in T2DM with preserved eGFR, which remind us to be alert of the decline of pulmonary function even in the early stage of DN.

## Supplementary information

**Additional file 1: Table S1.** Demographic, clinical characteristics and pulmonary function of control and T2DM subjects by smoking status

**Additional file 2: Table S2.** Demographic, clinical characteristics and pulmonary function of *T2DM subjects without or with albuminuria* by smoking status

## Data Availability

The data that support this study are available from the corresponding author only upon reasonable request, once the study has been published.

## Abbreviations

DN: Diabetic nephropathy
T2DM: Type 2 diabetes mellitus
UACR: Urine albumin-to-creatinine ratio
eGFR: Estimated glomerular filtration rate
CKD-EPI: Chronic kidney disease-Epidemiology collaboration
BMI: Body mass index
HbA1c: Glycated hemoglobin A1c
FBG: Fasting blood glucose
LDL: Low-density lipoprotein cholesterol
HDL: High-density lipoprotein cholesterol
TG: Triglyceride level
FVC: Forced vital capacity
FEV1: Forced expiratory volume in 1 second
TLC: Total lung capacity
DLCO: Diffusion capacity for carbon monoxide of lung
VA: Alveolar volume
OR: Odds ratio
CI: Confidence interval
