# Supplementary material for: Relationship between pulmonary function and albuminuria in type 2 diabetic patients with preserved renal function
Source: BMC Endocr Disord. 2020 Jul 23;20:112. doi: 10.1186/s12902-020-00598-1 (PMC7379808; doi:10.1186/s12902-020-00598-1)
Supplement: Supplementary file 1 — Additional file 1: Table S1. Demographic, clinical characteristics and pulmonary function of control and T2DM subjects by smoking status [file 12902_2020_598_MOESM1_ESM.docx]

**Table S1** Demographic, clinical characteristics and pulmonary function of control and T2DM subjects by smoking status

|  | Never smokers | |  | Former/Current smokers | |
| --- | --- | --- | --- | --- | --- |
|  | Control  （n=87） | T2DM  （n=156） |  | Control  （n=178） | T2DM  （n=170） |
| Sex |  |  |  |  |  |
| Male, n (%) | 27(31.0) | 61(39.1) |  | 165(92.7) ^ab^ | 161(94.7) ^ab^ |
| Female, n (%) | 60(69.0) | 96(60.9) |  | 13(7.3) | 9(5.3) |
| Age | 55.03 ± 11.50 | 54.56 ± 13.13 |  | 51.34 ± 9.16 ^ab^ | 52.55 ± 9.86 |
| BMI(kg/m^2^) | 25.21 ± 3.67 | 27.04 ± 3.91^a^ |  | 26.85 ± 3.50 ^a^ | 27.11 ± 3.82 ^a^ |
| HDL (mmol/L) | 1.31 ± 0.34 | 1.07 ± 0.27 ^a^ |  | 1.07 ± 0.28 ^a^ | 0.97 ± 0.22 ^abc^ |
| LDL (mmol/L) | 2.90 ± 0.81 | 2.85 ± 0.91 |  | 2.78 ± 0.80 | 2.71 ± 0.94 |
| TG (mmol/L) | 1.40 ± 0.81 | 1.93 ± 1.40 ^a^ |  | 2.31 ± 1.58 ^ab^ | 2.37 ± 1.70 ^ab^ |
| eGFR (ml/min/1.73m^2^) | 99.18 ± 12.51 | 105.51 ± 16.28 ^a^ |  | 101.72 ± 10.88 ^b^ | 105.42 ± 12.04 ^a^ |
| FVC%pred | 112.72 ± 14.09 | 105.48 ± 15.14 ^a^ |  | 104.52± 11.63 ^a^ | 100.29 ± 12.86 ^abc^ |
| FEV1%pred | 106.55 ± 14.36 | 100.86 ± 14.74 ^a^ |  | 99.13 ± 11.00 ^a^ | 95.95 ± 13.73 ^abc^ |
| FEV1/FVC (%) | 78.52 ± 4.85 | 79.21 ± 5.18 |  | 76.99 ± 4.83 ^ab^ | 77.59 ± 5.61 ^b^ |
| TLC%pred | 99.40 ± 11.04 | 93.89 ± 11.00 ^a^ |  | 96.55 ± 9.60 ^ab^ | 92.66 ± 9.71 ^ac^ |
| DLCOc%pred | 97.03 ± 14.91 | 91.39 ± 13.53 ^a^ |  | 93.71 ± 11.57 | 91.99 ± 13.57 ^a^ |
| DLCOc/VA%pred | 98.97 ± 16.68 | 100.38 ± 15.56 |  | 100.08 ± 12.62 | 102.73 ± 14.51 |

Data are presented as mean ± standard deviation, or number with percentage (%). ^a^*P* < 0.05 versus never smokers-control, ^b^*P* < 0.05 versus never smokers-T2DM, ^c^*P* < 0.05 versus former/current smokers-control

BMI: body mass index; LDL: low-density lipoprotein cholesterol; HDL: high-density lipoprotein cholesterol; TG: triglyceride level; eGFR: estimated glomerular filtration rate; FVC: forced vital capacity; FEV1: forced expiratory volume in 1 second; TLC: total lung capacity; DLCO: diffusion capacity for carbon monoxide of lung; VA: alveolar volume
