# Supplementary material for: Relationship between pulmonary function and albuminuria in type 2 diabetic patients with preserved renal function
Source: BMC Endocr Disord. 2020 Jul 23;20:112. doi: 10.1186/s12902-020-00598-1 (PMC7379808; doi:10.1186/s12902-020-00598-1)
Supplement: Supplementary file 2 — Additional file 2: Table S2. Demographic, clinical characteristics and pulmonary function of T2DM subjects without or with albuminuria by smoking status [file 12902_2020_598_MOESM2_ESM.docx]

**Table S2** Demographic, clinical characteristics and pulmonary function of T2DM subjects without or with albuminuria by smoking status

|  | Never smokers | |  | Former/Current smokers | |
| --- | --- | --- | --- | --- | --- |
|  | Without albuminuria（n=131） | Albuminuria  （n=25） |  | Without albuminuria（n=139） | Albuminuria  （n=31） |
| Sex |  |  |  |  |  |
| Male, n (%) | 50 (38.2) | 11 (44.0) |  | 132 (95.0) ^ab^ | 29 (93.5) ^ab^ |
| Female, n (%) | 81 (61.8) | 14 (56.0) |  | 7 (5.0) | 2 (6.5) |
| Age | 55.06 ± 12.72 | 51.96 ± 15.12 |  | 52.45 ± 10.02 | 53.00 ± 9.29 |
| BMI(kg/m^2^) | 26.96 ± 3.84 | 27.45 ± 4.30 |  | 26.99 ± 3.87 | 27.64 ± 3.59 |
| Duration (year) | 5.1 (0, 30) | 8.0 (0, 41) |  | 4.3 (0, 30) | 6.7 (0, 30) |
| HbA1c (%) | 9.16 ± 2.05 | 10.04 ± 2.43 |  | 8.73 ± 2.17 ^b^ | 9.42 ± 2.71 |
| FBG (mmol/L) | 7.41 ± 2.49 | 8.80 ± 3.77 ^a^ |  | 7.51 ± 2.50 ^b^ | 8.44± 2.75 ^a^ |
| HDL (mmol/L) | 1.07 ± 0.25 | 1.07 ± 0.37 |  | 0.97 ± 0.23 ^a^ | 0.97 ± 0.21 ^a^ |
| LDL (mmol/L) | 2.83 ± 0.88 | 2.98 ± 1.06 |  | 2.70 ± 0.92 | 2.77 ± 1.00 |
| TG (mmol/L) | 1.85 ± 1.41 | 2.38 ± 1.22 |  | 2.17 ± 1.35 | 3.25 ± 2.62 ^abc^ |
| eGFR (ml/min/1.73m^2^) | 105.58± 15.57 | 105.17 ± 19.93 |  | 105.39 ± 12.14 | 105.52 ± 11.74 |
| FVC%pred | 106.56 ± 15.20 | 99.84 ± 13.80 ^a^ |  | 101.46 ± 12.58 ^a^ | 95.05 ± 12.99 ^ac^ |
| FEV1%pred | 101.71 ± 14.95 | 96.40 ± 12.98 |  | 96.84 ± 13.19 ^a^ | 91.97 ± 15.56 ^a^ |
| FEV1/FVC (%) | 79.08 ± 5.12 | 79.87 ± 5.58 |  | 77.53 ± 5.33 ^ab^ | 77.89 ± 6.79 |
| TLC%pred | 94.45 ± 11.24 | 90.95 ± 9.30 |  | 93.16 ± 9.46 | 90.38 ± 10.62 ^a^ |
| DLCOc%pred | 92.17 ± 13.25 | 87.31 ± 14.52 |  | 93.05 ± 13.68 | 87.23 ± 12.13 ^c^ |
| DLCOc/VA%pred | 100.04 ± 14.72 | 102.21 ± 19.57 |  | 103.09 ± 14.98 | 101.09 ± 12.27 |

Data are presented as mean ± standard deviation, median (minimum, maximum), or number with percentage (%). ^a^*P* < 0.05 versus never smokers-without albuminuria, ^b^*P* < 0.05 versus never smokers-albuminuria, ^c^*P* < 0.05 versus former/current smokers-without albuminuria

BMI: body mass index; HbA1c: glycated hemoglobin A1c; FBG: fasting blood glucose; LDL: low-density lipoprotein cholesterol; HDL: high-density lipoprotein cholesterol; TG: triglyceride level; eGFR: estimated glomerular filtration rate; FVC: forced vital capacity; FEV1: forced expiratory volume in 1 second; TLC: total lung capacity; DLCO: diffusion capacity for carbon monoxide of lung; VA: alveolar volume
